# Supplementary material for: Stability of rhombohedral phases in vanadium at high-pressure and high-temperature: first-principles investigations
Source: Sci Rep. 2016 Sep 1;6:32419. doi: 10.1038/srep32419 (PMC5007533; doi:10.1038/srep32419)
Supplement: Supplementary Information [file srep32419-s1.pdf]

Supplementary information for

Stability of rhombohedral phases in vanadium at high-pressure and high-temperature: first-principles investigations

Yi X. Wang,[1,2] Q. Wu,[1] Xiang R. Chen,[2,*] and Hua Y. Geng[1,*]

[1]*National Key Laboratory of Shock Wave and Detonation Physics, Institute of Fluid Physics, CAEP; P.O. Box 919-102, Mianyang 621900, Sichuan, People's Republic of China.*

[2]*College of Physical Science and Technology, Sichuan University, Chengdu 610064, China.*

**e-mail: s102genghy@caep.ac.cn; xrchen@scu.edu.cn*

Lattice vibrations correction on the phase stability of vanadium. By using the finite temperature DFT method, we have investigated the thermo-electronic effects on the phase stability of vanadium in this work. It is an unusual approach to include electronic excitations but ignore the phonon contributions. We are indeed aware of the importance of lattice vibrations on phase stability of solid. But we also realized by empirical experiences that phonons' contribution is not large for some metals, and its impact on the phase diagram can be included via an approach similar to perturbation correction. In the case of vanadium we considered here, we knew that both phonon and thermo-electronic effects could affect the RH stability. The basis of our approach to ignore lattice vibrations actually relys on Landa's phonon calculation (Ref. 21 of the main text), which demonstrated that phonons' impact on RH stability is not big. We took this as an assumption and calculated the thermo-electronic effects separately. There are two cases one could expect: (i) the thermo-electronic effects are at the comparable level with the lattice vibrations, thus invalidates our assumption that phonons contribution is negligible, and a full consistent treatment containing both lattice vibrations and electronic excitations is required; and (ii) the thermo-electronic effects are much more stronger, and validates our assumption that phonons

contribution can be treated as a perturbation. Comparing our thermo-electronic results and Landa's lattice dynamics confirms that the case (ii) is true. For many metals, thermo-electronic effects are usually smaller than phonons contribution. But our discovery here is that for vanadium, it is opposite. In order to eliminate the worry about the validation of our conclusion, we present a simple assessment of the phonons contribution below.

Starting from the definition of the Helmholtz free energy of a solid

$$F(V,T)=E_c(V)+F_e(V,T)+F_{vib}(V,T). \tag{1}$$

in which E_c stands for cold energy, F_e stands for the free energy from thermal electrons and F_{vib} stands for the lattice dynamics contribution, respectively. The phase diagram given in Ref. 21 implies that at the given phase boundary between BCC and RH phase at 182 GPa and 8000 K, there is

$$E_{c,RH}-E_{c,BCC}=\Delta E_c=F_{vib,BCC}-F_{vib,RH}=-\Delta F_{vib}(T=8000\mathrm{K}). \tag{2}$$

On the other hand, if including the thermo-electronic effects only, our finite temperature phase diagram implies

$$\Delta E_c=F_{e,BCC}-F_{e,RH}=-\Delta F_e(T\approx 2000\mathrm{K}). \tag{3}$$

Since the Debye temperature of vanadium is about 326 K at the ambient conditions. Compression to high pressures about 200 GPa increases the Debye temperature to a level of 600 K, but is still far smaller than the temperature scale we are considering here. This implies that we can use the high temperature expansion to approximate the lattice vibrational free energy F_{vib}. Namely

$$F_{vib}(T)=-3k_BT\left[\ln\left(\frac{T}{\theta_0}\right)-\frac{1}{40}\left(\frac{\theta_2}{T}\right)^2+\cdots\right]\approx -3k_BT\ln\left(\frac{T}{\theta_0}\right). \tag{4}$$

Therefore

$$-F_{vib}(T)=-3k_BT\ln\left(\frac{\theta_{0,RH}}{\theta_{0,BCC}}\right). \tag{5}$$

and

$$\frac{F_{vib}(T=8000\mathrm{K})}{F_{vib}(T=2000\mathrm{K})}=4. \tag{6}$$

This gives an estimation of the lattice free energy difference at 2000 K, which is about one fourth of that at 8000 K, and thus about one fourth of $\Delta F_e(T = 2000\text{K})$. It is evident that neglect of the lattice vibrational contribution overestimates the transition temperature. An approach to correct this and include the phonon effect on the phase boundary is to consider the free energy of thermal electrons as a function of temperature. For most metals, the free electron gas model can be applied and the electronic free energy is

$$F_e = -\frac{1}{2}\Gamma T^2, \tag{7}$$

in which Γ is a constant. Thus $\Delta F_e \propto T^2$. At the real transition temperature T', one has

$$\Delta F(T') = \Delta E_c + \Delta F_e(T') + \Delta F_{vib}(T') = 0. \tag{8}$$

Approximate $\Delta F_{vib}(T')$ by $\Delta F_{vib}(T = 2000\text{K})$, one gets

$$-\Delta F_e(T') \approx -\frac{3}{4}\Delta F_e(T = 2000\text{K}). \tag{9}$$

Eq. (7) then gives

$$T' \approx \sqrt{\frac{3}{4}} \times 2000\text{K} = 1732\text{K}, \tag{10}$$

or

$$\frac{T'-T}{T} = \frac{\Delta T}{T} = 0.13. \tag{11}$$

It shows that if include only the thermal electrons and neglect lattice vibrations, the phase transition temperature will be overestimated by about 13% (or ~268 K at ~200 GPa). This confirms the argument that the stability of RH will be reduced further by phonon effects. But the correction magnitude is actually small. A version of the corrected phase diagram is given in the Fig. S1. The concern that including both thermo-electron effects and phonon contribution might greatly alter the phase diagram should not exist.

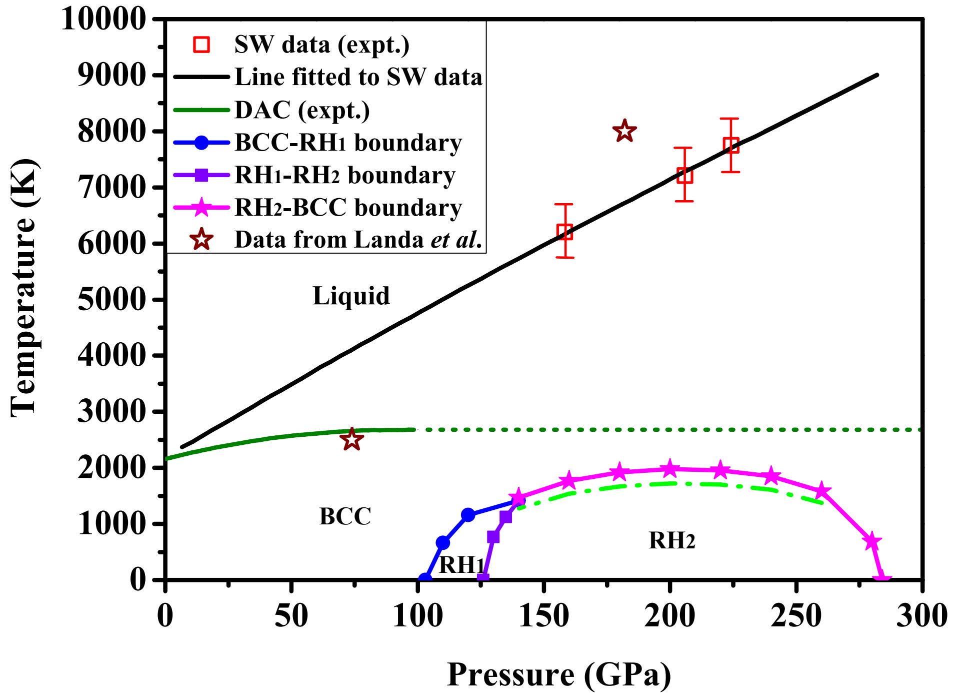

Figure S1. (Color online) Phase diagram of vanadium at high pressure and finite temperature as shown in the Fig. 6 of the main text. The dash-dotted line indicates the correction of lattice dynamics if included via a simple assessment.
